# Nerve Growth Factor (NGF) Encourages the Neuroinvasive Potential of Pancreatic Cancer Cells by Activating the Warburg Effect and Promoting Tumor Derived Exosomal miRNA-21 Expression

**DOI:** 10.1155/2022/8445093

**Published:** 2022-10-15

**Authors:** Tao Peng, Yao Guo, Zheng Gan, Yan Ling, Jiongxin Xiong, Xueyi Liang, Jing Cui

**Affiliations:** ^1^Department of Pancreatic Surgery, Union Hospital, Tongji Medical College, Huazhong University of Science and Technology, Wuhan, Hubei 430022, China; ^2^Department of Anesthesiology, Union Hospital, Tongji Medical College, Huazhong University of Science and Technology, Wuhan, Hubei 430022, China; ^3^Medical Examination Center, Union Hospital, Tongji Medical College, Huazhong University of Science and Technology, Wuhan, Hubei 430022, China

## Abstract

**Background:**

It has been reported that signaling from the nerve growth factor (NGF) pathway associated with peripheral nerves is able to contribute to perineural invasion (PNI) of pancreatic cancer (PC). Nevertheless, the underlying mechanism by which NGF leads to PNI remained poorly understood.

**Methods:**

Western blotting was employed to determine NGF level in PC and paracarcinoma tissues and in PC cell lines as well as pancreatic ductal epithelial cells. MiaPaCa-2 and CFPAC-1 cells were treated with 100 ng/ml of NGF or the NGF inhibitor Tanezumab for 24 h, CCK-8 and Transwell assays were employed to test cell proliferation, invasion, and migration, respectively. TrkA expression was knocked down in MiaPaCa-2 and dorsal root ganglion (DRG) cells treated with NGF to determine its effect on the Warburg effect. To reveal that the NGF-TrkA signaling pathway was closely associated with PC PNI, *in vitro* neuroinvasion model was established by using MiaPaCa-2 cells via coculturing DRG cells in Matrigel. Further, exosomes were extracted from PC cells and identified by examining the levels of specific markers for exosomes. Then RT-qPCR was applied to test miR-21-5p level in tumor derived exosomal (TDE-miR-21-5p). RIP assay was performed to validate NGF and miR-21 binding ability in MiaPaCa-2 cells. Rescue experiments were performed by using coprocessing of Tanezumab and miR-21-5p mimic on MiaPaCa-2 cells, followed by coculture with DRG cells. Subsequently, we used a model of neuroinvasion in nude mice to assess the effect of NGF *in vivo* on tumor nerve invasion as well as on nociceptive transmission.

**Results:**

NGF level was preeminently higher in PC tissues and cell lines than in paracarcinoma tissues and normal pancreatic epithelial cell lines. NGF promoted MiaPaCa-2 and CFPAC-1 cell invasion and migration, while Tanezumab treatment showed the opposite results. Besides, NGF binding to TrkA receptors encouraged the intracellular Warburg effect in PC and DRG cells. TrkA blocking-up could restrain NGF induced PC cell migration and neural invasion. Mechanistically, NGF could upregulate TDE-miR-21-5p levels, and DRG cells took up TDE to activate the Warburg effect and stimulate nociceptor gene expression. miR-21-5p inhibitor could abolish the facilitative effect of NGF on PNI in MiaPaCa-2 cells. *In vivo* tumorigenesis experiments, Tanezumab markedly alleviated nerve invasion of PC cells as well as relieved nociceptive conduction in animal models.

**Conclusions:**

These findings displayed that NGF/TrkA encouraged the neuroinvasive potential of PC cells by activating the Warburg effect in DRG cells through upregulation of TDE-miR-21-5p expression.

## 1. Introduction

Pancreatic cancer (PC) is a relatively common malignancy of the digestive system and one of the human malignancies with the worst prognosis [1]. Approximately 213,000 people worldwide die from PC each year [2]. Its pathogenesis remains unknown, and it is currently only well established that family history, smoking, diabetes mellitus, obesity as well as alcohol abuse are high risk factors for PC [3]. PC has an insidious onset and often has no obvious clinical manifestation in the early stage. However, the disease progresses more rapidly, and until the clinical symptoms appear or the diagnosis is confirmed, most cases lose the opportunity of surgical treatment because of local invasion or hematogenous, lymphatic, and distant metastasis. Surgery is accessible to only less than 20% of patients, with an additional 70% of cases being amenable to only palliative surgery [4]. Even radical surgical cases that achieve clinicopathological cure criteria, curative effects remain unsatisfactory.

Infiltration versus metastasis is one of the most characteristic biological properties of tumors [5]. Generally, it is believed that there are four main pathways between tumor invasion and metastasis, including local invasion, blood, lymphatic, and seeding metastasis [6]. In addition to these four pathways, tumors can undergo infiltration around nerve fibers and metastasis along nerves, that is, perineural invasion (PNI), which refers to the phenomenon of perineural invasion by tumor cells filling the perineurial space, wrapping around nerves in a continuous concentric sheath-like pattern, infiltration, and metastasis of extension along nerves around nerve fibers or into perineurium within the perineurium [7]. Although this more specific biological behavior is seen in biliary tract tumors, PC, parotid gland tumors, prostate cancer, and breast cancer, as well as basal and squamous cell carcinomas of the head and neck, perineural invasion is more common in PC. The fact that pancreatic tissue itself is rich in neural tissue determines the predisposition of PC to perineural invasion, which also constitutes the anatomical basis for the predisposition of pancreatic cancer to perineural tissue invasion. Perineural invasion, which starts within the pancreas, evolves to perineural invasion of the pancreas and subsequent metastasis to distant sites such as retroperitoneum, which is also thought to be an important cause of surgical margin remnant and persistent extra-pancreatic dissemination [8]. In the PNI of PC, a variety of genes or proteins are involved in the process of promoting tumor cell growth and inhibiting their apoptosis. Their interaction creates a favorable microenvironment for the invasiveness and neurotropic growth of tumor cells. Special growth factors produced by nerves or the cells associated with them are important reasons for promoting the growth and survival of cancer cells with a proneural nature [9].

Nerve growth factor (NGF) plays a very important role in this behavior. NGF, an important neuropeptide in the neurotrophin family with a molecular weight of about 140 KD, is composed of three peptide chains, *α*, *β*, and *γ*, bound noncovalently at the ratio of *α*2*βγ*2. Subunit *β*, which consists of an identical skin chain containing 188 amino acid residues each, is the active zone of NGF and the only of the three subunits to have the biological activities of NGF. Under normal circumstances, NGF exerts regulatory effects on neuronal survival, growth and development, differentiation, regeneration, and functional maintenance. Numerous studies have shown that high level of NGF in PC are closely associated with tumor cell migration and perineural invasion. For example, NGF activated the ERK1/2 signaling pathway in PC by regulating CD133 to accelerate cell migration and invasion [10]. Further, tyrosine kinase receptor A (TrkA) is the functional receptor for NGF and when bound to NGF can activate tyrosine kinase signaling systems that initiate cellular activity to produce biological effects. NGF and TrkA are its high affinity receptors that play a crucial role in cancer pathogenesis through cell proliferation, angiogenesis, invasion, and migration. Vera et al. [11] demonstrated that NGF/TrkA could promote the malignant behavior of ovarian cancer cells by upregulating oncogenic proteins such as vascular endothelial growth factor and c-myc expression during ovarian cancer progression. Further, NGF acts by binding to receptors on the surface of target cells, which are divided into a high affinity TrkA and a low affinity receptor p75. NGF/TrkA facilitated PC cell proliferation and invasion in coculture system by activating the PI3K/Akt/GSK signaling cascade [12]. However, the specific mechanism by which the NGF/TrkA pathway leads to PNI in PC remains to be further explored.

Tumor cells consume far more glucose than normal cells. More importantly, even under aerobic conditions glucose in tumor cells is not oxidized exhaustively but is broken down to produce lactate, a phenomenon that was discovered by the German biochemist O.H. Warburg and is therefore called the Warburg effect [13]. Although the capacity rate of glycolysis is lower than that of oxidative phosphorylation, glycolysis can meet the energy demand of rapid tumor cell growth [14]. Moreover, high levels of glycolysis within tumor cells would consume a large amount of glucose in the tumor microenvironment and lead to the accumulation of lactate, which in turn inhibits lymphocytes and tumor infiltration [15]. An increasing number of studies have shown that an excessive level of glycolysis is closely associated with PC progression and invasion [16]. The molecular mechanism by which cancer cells achieve metabolic reorganization is still unclear. Therefore, elucidating the molecular mechanism of metabolic reorganization of tumor cells will provide a new and effective way for tumor targeted therapy.

In this study, an *in vitro* model generated by coculturing mouse dorsal root ganglion (DRG) neuronal cells and PC cells, and an *in vivo* PNI model were applied to investigate the function and underlying mechanisms of NGF/TrkA signaling in the progression and pathogenesis of PNI, contributing to the development of therapeutic regimens for PC.

## 2. Materials and Methods

### 2.1. Patients and Clinical Samples

A total of 90 cases were selected and included at the Union Hospital attended patients with PC. The pathological diagnosis of PC was carried out according to the standards of the World Health Organization. Written informed consent was signed by each patient.

### 2.2. Cell Lines and Culture

Panc-1, AsPc-1, MiaPaCa-2, BxPC-3, Capan-2, CFPAC-1, and HPDE cell lines were obtained from Shanghai Huiying biotech Co., Ltd, Shanghai, China. Subsequently, cells were subjected to mycoplasma testing, isoenzyme testing, and cell viability assays were performed by the biological company (GeneCreate Biological Engineering Co., Ltd., Wuhan, China). All cells were cultured in DMEM medium containing 10% fetal bovine serum, 100 U/mL penicillin and 100 *μ*g/mL streptomycin. All cell lines were maintained at 37°C in a humidified incubator with 5% CO_2_. MiaPaCa-2 and CFPAC-1 cells were treated with 100 ng/ml NGF or NGF inhibitor Tanezumab for 24 hours, and cells were collected for further experiments.

### 2.3. Genetic Overexpression and Knockdown

The shRNAs were designed by Qiagen to knock down TrkA. The miR-21-5p mimic and miR-21-5p inhibitor, and corresponding controls NC mimic and NC inhibitor were purchased from Geneseed (Shanghai, China). TrkA shRNA constructs were produced using the following target sequences: 5′- GCTTGGCTGATACTGGCATCT-3′ for sh70 and 5′-ACCTGACTGAGCTCTACATCG-3′ for sh200. Stable infections were performed for lentiviral constructs: plv-hu6-EF1a-puro-negative control shRNA, plv-hu6-TrkAsh70, and plv-hu6-EF1a-puro-TrkAsh200. Confluent cells were diluted in DMEM medium, and the cells were observed to grow to about 70% confluence when the cell monolayer was covered with serum-free DMEM medium. The plasmid transfection was performed by using Lipofectamine®3000 transfection reagent, all cells in each group were collected for subsequent experiments after incubating in an incubator at 37°C with 5% CO_2_ for a specified period of times.

### 2.4. RNA Extraction and PCR Assays

The cells from each group after transfection were collected, and Trizol (ThermoFisher, USA) was added to extract total cellular RNA according to the instructions of total RNA extraction reagent. After measurement of A260/A280 between 1.8-2.0. Total cellular RNA was reverse transcribed into cDNA using a reverse transcription Kit (Qiagen, Germany), with a 20 *μ*L of reverse transcription reaction system including 0.2 *μ*L of MMLV (200 U/*μ*L), 1.2 *μ*L of miR-RT primer (1 *μ*mol/L), 0.75 *μ*L of dNTP (10 mmol/L), 4 *μ*L of 5 × RT buffer, 10 *μ*L of total RNA and 3.85 *μ*L of RNase free ddH2O. The reaction conditions were as follows: 37°C for 30 min and 85°C for 10 min. PCR products were detected with steponeplus real time PCR system (Thermo Fisher, USA). The real time PCR reaction system was 20 *μ*L, including 1 *μ*L of cDNA template, 0.5 *μ*l each of upstream and downstream primers (10 *μ*mol/L), 10 *μ*L of SYBR GREEN mastermix, and 8 *μ*L of RNase free ddH2O. GAPDH and U6 served as internal references. The relative expression levels were calculated by using 2^−ΔΔCT^ method. The efficiency of the PCR should be between 90-110% (3.6 > slope > 3.1). miR-21-5p forward, 5′- GCC GCT AGC TTA TCA GAC TGA TGT -3′; and reverse, 5′- CGA CAG TGG GAG TGA CGC CCT TA -3′; NGF forward: 5′- CTG GCC ACA CTG AGG TCG AT-3′; and reverse, 5′- TCC TGC AGG GAC ATT GCT CTC-3′. U6 forward, 5′- TGCGGGTGCTCGCTTCGGCAGC-3′; and reverse, 5′- CCA GTG CAG GGT CCG AGG T-3′; GAPDH forward, 5′- GCA CCG TCA AGG CTG AGA AC -3′; and reverse, 5′- TGG TGA AGA CGC CAG TGG A -3′.

### 2.5. Western Blotting

Total cell protein was extracted with RIPA lysate, and the protein concentration was determined using a BCA protein assay kit (Thermo Fisher Scientific, Waltham, MA, USA) in a microplate reader. After denaturation for 10 min with the addition of loading buffer, 50 *μ*g of protein samples were subjected to SDS-PAGE and transferred onto PVDF membranes. The membrane was blocked with blocking solution (5% nonfat dry milk) for 2 h and subsequently washed three times using TBST. Specific primary and secondary antibodies were next added separately, followed by incubation on a shaker. ImageJ software was applied to detect and analyze the gray values of protein bands on the membrane. The following primary antibodies: *β*-actin (1 : 1000, Abcam, ab8227, Cambridge, UK), NGF (1 : 1000, ab52918, Abcam), TrkA (1 : 1000, ab109010, Abcam), PI3K (1 : 1000, ab32089, Abcam), p-AKT (phospho T308) (1 : 500, ab38449, Abcam), AKT (1 : 1000, ab8805, Abcam), mTOR (1 : 1000, ab109268, Abcam), p-mTOR (phospho S2481) (1 : 1000, ab137133, Abcam), CD63 (1 : 1000, ab134045, Abcam), TSG101 (1 : 1000, ab125011, Abcam), ALIX (1 : 500, ab88388, Abcam), CD9 antibody (1 : 2000, ab92726, Abcam), TRPV1 (1 : 1000, ab6166, Abcam).

### 2.6. Glucose, Lactate, Adenosine Triphosphate (ATP) Levels, and Extracellular Acidification Rate (ECAR)

The medium was collected after cell transfection. Simply put, glucose levels are measured with a glucose test kit (Thermo Fisher, USA). The lactic acid content was measured with a lactic acid meter (Thermo Fisher, USA). The content of ATP was determined using the Cell Titer-Glo Cell Viability Assay (Thermo Fisher, USA). ECAR was determined using the Seahorse XF96 Cell Efflux Analyzer according to the manufacturer's instructions.

### 2.7. Exosome Isolation and Identification

Exosomes were isolated by precipitation using exoquick reagent (SBI) according to the manufacturer's instructions. Briefly, conditioned medium was incubated with exoquick reagent (5 : 1) for 12 h or longer, followed by centrifugation at 1500 g for 30 min, and the precipitated exosomes were suspended in 100 mL of PBS, then stored at -80°C. In addition, total exosome isolation (Magen) was performed using serum exosomes. Reagents were added to serum samples and incubated at 4°C for 30 min, followed by centrifugation of 1 ml of serum samples at 2000 g for 30 min to remove cells and debris. Alternatively, exudates observed by transmission electron microscopy were suspended in glutaraldehyde, dripped onto carbon-containing copper mesh and stained with 2% uranyl acetate, then dried and imaged.

### 2.8. Co-Immunoprecipitation (co-IP)

MiaPaCa-2 and DRG cells were lysed with RIPA buffer (Thermo Fisher, USA), and the supernatant was collected and incubated with NGF and TrkA antibodies overnight at 4°C. This mixture was incubated with 100 microliters of A/G agarose beads overnight at 4°C. Then, the agarose sphere-antigen-antibody complexes were collected by transient centrifugation and washed 3 times with PBS. Next, the complex with protein-added buffer for 5 minutes. Intracellular interacting proteins were analyzed by immunoblotting technique.

### 2.9. RNA Immunoprecipitation (RIP) Assay

Protease inhibitor EDTA as well as RNase inhibitor (Qiagen, Hilden, Germany) were added to IP lysis buffer to lyse cells. After the addition of magnetic beads preclearing for 30 min, IgG antibody and NGF antibody (Abcam, USA) were added, respectively, and then the added magnetic beads were rotated to mix for 2 h at room temperature. Aspirate supernatant on magnetic stand and wash beads with IP lysis buffer. After removal of proteins by addition of proteinase K, Trizol LS was added to extract RNA and finally subjected to subsequent analysis.

### 2.10. Assessment of Neural Cancer Cell Interactions

PNI model was used to simulate the tumor and surrounding neural microenvironment. DRGs were placed on ice in DMEM in 24 wells containing 25 *μ*L of Matrigel at a distance of approximately 1 mm from tumor colonies suspended in 25 *μ*L of matrix. To rule out nonspecific metastasis of tumor cells, another 25 microliters of DRG-free male basement membrane was placed on its back. The incubator was placed in an incubator at 37°C and saturated with 5% CO_2_ for 20 minutes in a humid environment to perform Matrigel polymerization. After solidification, the DMEM medium was added every 2 days. The cell suspensions on adjacent sides were photographed by using an inverted microscope technique. To quantitatively study the growth of tumor cells, we determined the tumor boundary and tumor boundary as the *γ* parameter, the tumor cell metastasis distance as the *α* parameter, and the direction of the DRG as the tumor growth length. Invasion index was *α*/*γ*, and DRG growth index was *β*/*γ*. The moving distance is performed by the image analysis software of the microscope imaging system (NIS-Elements, Nikon Instruments Inc., China).

### 2.11. Cell Migration and Invasion Assay

The ability of cell invasion and migration was detected by treatment with 8.0-*μ*m chamber plates. The upper surface of the Transwell filter we used was coated with or without Matrigel (BD, New Jersey, USA). Firstly, cells were planted into the 8.0 *μ*m chamber plates, then 300 *μ*L of serum-free DMEM medium was added to the upper compartment of the chamber, and then 500 *μ*L of DMEM medium supplemented with 10% FBS was added to the lower chamber for 48 h incubations. Then, the noninvasive cells on the upper side of the chamber were suspended with a cotton swab, and then the invasive cells were fixed in 4% paraformaldehyde and stained with a crystal violet solution. We stained infiltrating cells by using an Olympus IX70 inverted microscope (Olympus Corp, Tokyo, Japan) and randomly selected the best six fields of view, and each experiment was repeated three times.

### 2.12. Cell Proliferation Assay

The treated cells from each group were seeded in 96 well culture plates at a cell number per well of 3 × 103, and 5 replicate wells were set in each group. Cell culture supernatants were removed at 0 h, 24 h, 48 h, and 72 h after seeding, and 10 *μ*l of CCK-8 solution was added to each well according to the CCK-8 cell proliferation activity assay kit instructions (Sigma-Aldrich, USA). After incubation at 37°C in 5% CO2 for 1 h, the OD value at 450 nm was measured by using a microplate reader (Bio-Rad, USA), and the cell growth curve was plotted.

### 2.13. Immunohistochemistry

S-100, CK19, and NGF were detected by immunohistochemistry by using SABC kit according to the manufacturer's requirements. Briefly, incubate with S-100 (1 : 100), NGF (1 : 50) primary antibodies overnight, with an appropriate biotin secondary antibody for 30 min at room temperature, then with streptavidin peroxidase Incubation (Dako LSAB + HRP kit). Slides were stained with DAB and counterstained with hematoxylin.

### 2.14. *In Vivo* PNI Model

Animal experiments were approved and supervised by the Animal Ethics Committee of Huazhong University of Science and Technology. 5 week old male athymic BALB/C nude mice were obtained from Experimental animal center of Huazhong University of Science and Technology, and subsequently randomly divided into 2 groups, including PNI + PBS and PNI + Tanezumab. MiaPaCa-2 cell suspension was injected subcutaneously into the midline of the back of the mice. Tanezumab was then administered at 10 mg/kg/kg/d, and tumors were excised within 8 weeks and histologically examined to determine neural PNI. In order to evaluate the role of NGF in tumors that invade along nerves, the nerve invasion model is basically performed in this way. Under the microscope, MiaPaCa-2 cells were injected into the sciatic nerve of paralyzed rats separated from the tibial and peroneal nerve bifurcations. 3 ml of normal saline was injected into the right sciatic nerve as a sham-operated control group. 3 microliters of cytoplasm were microinjected with a 5 microliter microsyringe at a concentration of 1 x 10^5^ cells per microliter. In order to evaluate the function of the sciatic nerve innervating the paw muscles of the hindlimbs, we measured the following parameters: rough movements, 10 minutes per week, and repeated bite marks on the hindlimbs; limb function, according to the degree of response of the hindlimb paws to manual stretching, divided into 4-1 (total paw paralysis), sciatic nerve function index, the unit of elongation (mm) between the 1st and 5th toes of the hind legs of mice, 7 weeks per week.

### 2.15. Statistical Analysis

SPSS 22.0 and GraphPad Prism 7.0 were used for data analysis and mapping. The pairwise comparisons were analyzed using the chi-square test. The measurement data were represented as mean ± SEM with normal distribution and homogeneity of variance. Student's *t*-test was performed for the comparison between two groups. The means of the different groups were compared using one-way or two-way analysis of variance (ANOVA) following Tukey's post hoc test. *P* < 0.05 was considered as statistically significant difference. All experiments were repeated 3 times (*n* = 3).

## 3. Results

### 3.1. NGF Indicated the Unfavorable Prognosis of PC Patients

**Figure 1:**
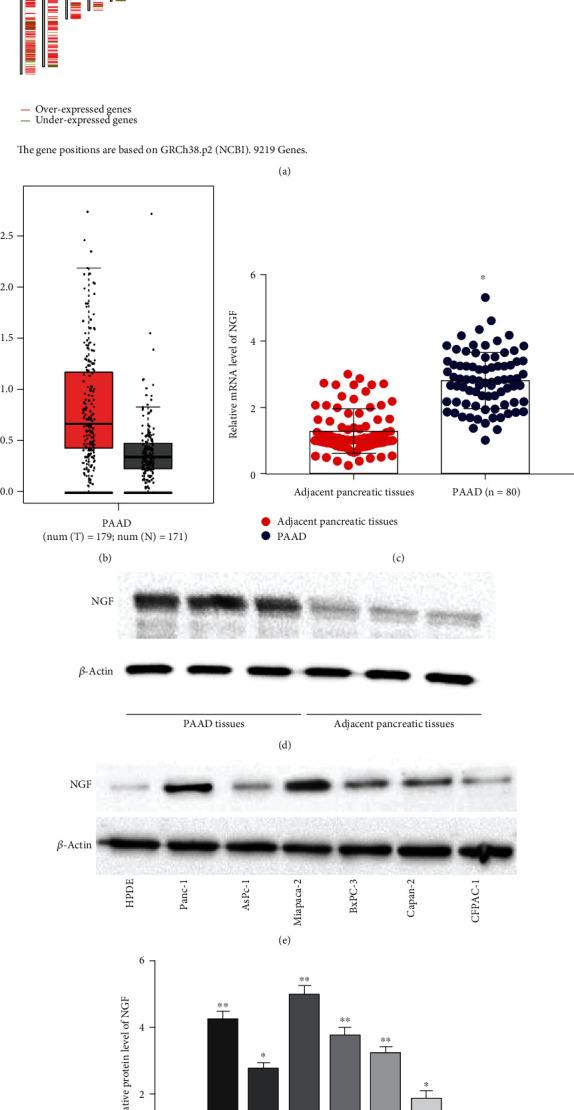
NGF was increased in PC tissues and cell lines. (a) Differentially expressed genes on chromosomes in PC. (b) GEPIA database analysis of NGF expression in PC samples and normal samples. (c) NGF level in PC tissues and adjacent tissue samples collected from 80 patients was tested by RT-qPCR. ^∗∗^*P* < 0.01, unpaired *t*-test. (d) Differential of NGF was assessed by Western blotting in three randomly selected pairs of 80 samples. (e-f) Relative level of NGF in PC cell lines (Panc-1, AsPc-1, MiaPaCa-2, BxPC-3, Capan-2, CFPAC-1) and pancreatic ductal epithelial cells HPDE was tested by Western blotting. Data were presented as mean ± SEM. *N* = 3, ^∗^*P* < 0.05 compared with adjacent pancreatic tissues. ^∗∗^*P* < 0.01 compared with adjacent pancreatic tissues or HDPE.

To explore the potential involvement of NGF in PC, we analyzed the differential level of NGF in PC samples with the aid of a bioinformatics.  Figure 1(a) was shown as differential genes on chromosomes in the PC samples, and the differential expression genes on chromosome 1, where NGF was located, were found to be most enriched.  Figure 1(b) revealed that NGF was prominently higher in pancreatic cancer than in normal samples. Subsequently, we performed RT-qPCR to determine the level of NGF in 80 paired PC tissues and matched with normal tissues, and three groups of samples were randomly selected to detect the NGF protein expression level by using western blotting. As shown in Figure 1(c)–(d), the NGF level was higher in tumor tissues of PC patients than that in the corresponding normal tissues. Similarly, NGF was overexpressed in human PC cell lines compared with that of normal pancreatic epithelial cell lines, especially in MiaPaCa-2 cells (Figure 1(e)–1(f)).

### 3.2. NGF Promoted PC Cell Migration and Invasion In Vitro

**Figure 2:**
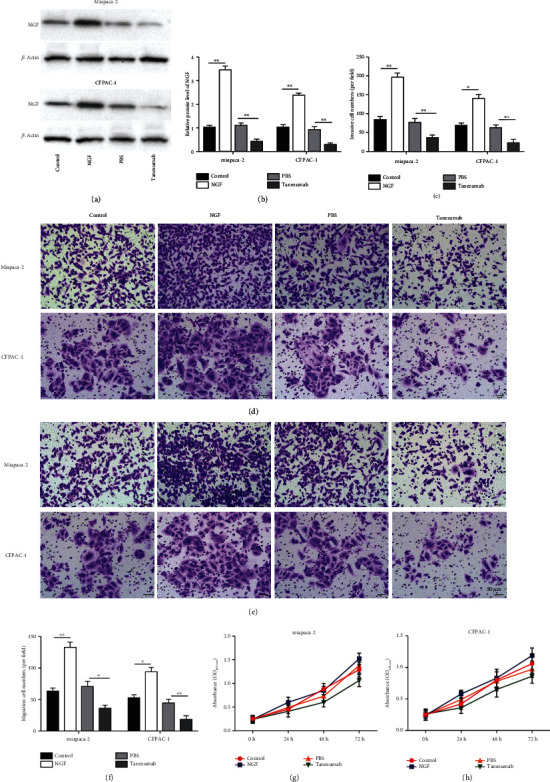
NGF promoted PC cell migration and invasion *in vitro.* MiaPaCa-2 and CFPAC-1 cells were treated with 100 ng/ml of NGF or the NGF inhibitor Tanezumab for 48 h. (a-b) Western blot analysis was employed to test the level of NGF in Miapaca-2 and CFPAC-1 cells treated with NGF or the NGF inhibitor Tanezumab. (c-f) Transwell assay was employed to test migration and invasion of MiaPaCa-2 and CFPAC-1 cells treated with NGF or the NGF inhibitor Tanezumab. (g-h) CCK8 was used to detect cell proliferation of MiaPaCa-2 and CFPAC-1 cells treated with NGF or the NGF inhibitor Tanezumab. *N* = 3, ^∗^*P* < 0.05, ^∗∗^*P* < 0.01.

Next, we sought to analyze the effect of NGF on the proliferation and invasive capacity of PC cells. MiaPaCa-2 and CFPAC-1 cells were treated with 100 ng/ml of NGF or the NGF inhibitor Tanezumab for 24 h. The Western blotting results revealed that NGF level was effectively modulated in MiaPaCa-2 and CFPAC-1 cells (Figure 2(a)–2(b), *p* < 0.01). As shown in Figures 2(c)–2(f), NGF promoted MiaPaCa-2 and CFPAC-1 cell invasion and migration, while Tanezumab treatment inhibited cell invasion and migration. Differently, we found that NGF or Tanezumab treatment had insignificant effects on cell proliferation (Figure 2(g)–2(h), *p* > 0.05). These results suggest that NGF functions by promoting migration and invasion of PC cells.

### 3.3. NGF Binding to TrkA Receptors Encouraged the Intracellular Warburg Effect in PC Cells and DRG Cells

**Figure 3:**
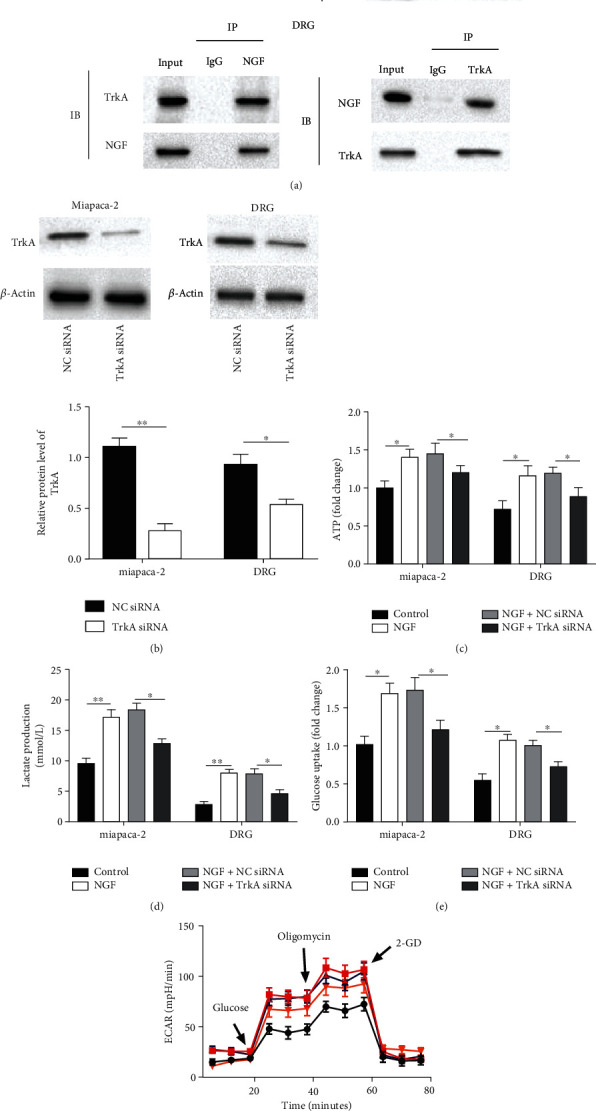
NGF binding to TrkA receptors encouraged the intracellular Warburg effect in PC cells and DRG cells.

It has been reported that Warburg effect is able to participate in regulating neural function [17]. First, we detected and validated NGF and TrkA receptor binding in PC and DRG cells by using protein co-immunoprecipitation (Figure 3(a)). Subsequently, to understand the role of NGF-TrkA signaling in PC and DRG cells, we knocked down TrkA expression in MiaPaCa-2 and DRG cells treated with NGF to determine its effect on the Warburg effect. The efficiency of TrkA siRNA on its level in PC and DRG cells was confirmed by Western blotting (Figure 3(b), *p* < 0.01). Glucose uptake analysis, lactate production analysis, and ATP analysis discovered that NGF overexpression increased the glucose uptake, lactate production, and ATP accumulation. Further, TrkA knockdown partially reversed the facilitative effects of NGF on glucose uptake, lactate production, and ATP accumulation (Figure 3(c)–3(e), *p* < 0.05). ECAR analysis discovered that NGF promoted the glycolytic capacity, which was reversed by TrkA inhibition (Figure 3(f), *p* < 0.01). In short, these findings revealed that NGF could encourage the Warburg effect of PC and DRG cells.

We knocked down TrkA expression in MiaPaCa-2 and DRG cells treated with 100 ng/ml of NGF for 48 h to determine its effect on the Warburg effect. (a) Co-IP was employed to test the interaction between NGF and TrkA in MiaPaCa-2 and DRG cells. (b) Western blotting was employed to test the level of TrkA in MiaPaCa-2 DRG cells transfected with TrkA siRNA. (c-e) Glucose uptake, lactate production, and ATP assays were employed to test glucose uptake, lactate production, and ATP accumulation in MiaPaCa-2 DRG cells treated with 100 ng/ml of NGF or/and transfected with TrkA siRNA for 48 h. (f) Extracellular acidification rate (ECAR) was employed to test and detects the glycolytic capacity of DGR cells treated with 100 ng/ml of NGF or/and transfected with TrkA siRNA for 48 h. *N* = 3, ^∗^*P* < 0.05, ^∗∗^*P* < 0.01.

### 3.4. NGF/TrkA Activated the PI3K/AKT/mTOR Signaling Pathway and Enhanced the Interaction between PC Cells and DRG Cells

**Figure 4:**
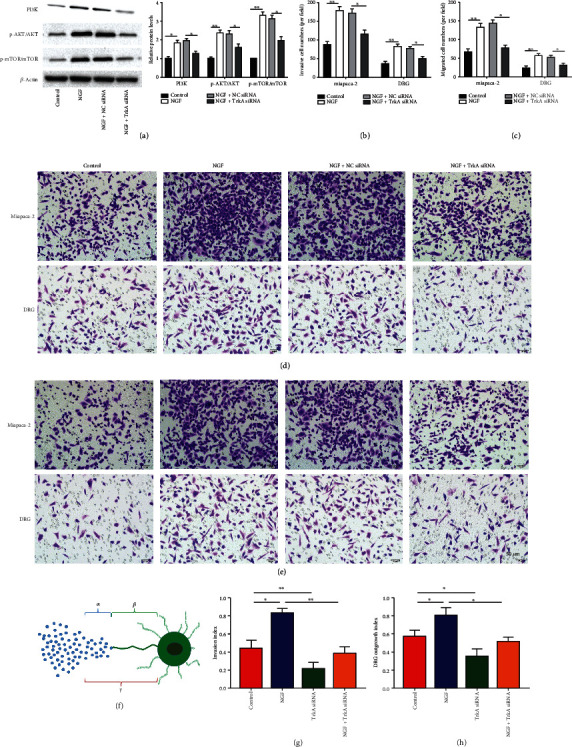
NGF/TrkA activated the PI3K/AKT/mTOR signaling pathway and enhanced the interaction between PC cells and DRG cells. (a)Western blotting was employed to test the levels of PI3K, p-AKT, and p-mTOR in MiaPaCa-2 cells treated with 100 ng/ml of NGF or/and transfected with TrkA siRNA for 48 h. (b-e) Transwell was used to detect migration and invasion of DGR or MiaPaCa-2 cells treated with 100 ng/ml of NGF or/and transfected with TrkA siRNA for 48 h. (f) Schematic of MiaPaCa-2 and DGR invasion. (g) DRG growth index. (h) Neuroinvasion index. *N* = 3, ^∗^*P* < 0.05, ^∗∗^*P* < 0.01.

To investigate potential regulators of NGF/TrkA signaling that induce cell invasion and migration, we focused our attention on downstream pathways. We used western blot assay to detect PI3K/AKT/mTOR pathway-related proteins. The results demonstrated that PI3K, p-AKT, and p-mTOR were upregulated in NGF overexpression PC cells, whereas transfection of TrkA siRNA reversed the results (Figure 4(a), *p* < 0.01). Moreover, PC and DRG cell migration and invasion numbers were increased after NGF treatment, and this result was partially counteracted by TrkA downregulated (Figure 4(b)–4(e), *p* < 0.01). Signaling through the NGF-TrkA pathway was involved not only in PC cell growth but also in the process of PNI in PC cells [18]. To confirm whether the NGF/TrkA axis is involved in the PNI of PC cells, a model of neuroinvasion was constructed *in vitro* by culturing rat DRG cells and MiaPaCa-2 cells using a Matrigel coculture system. The invasion schematic is shown in Figure 4(f). The results revealed that overexpression of NGF memorably increased the MiaPaCa-2 invasion index and DRG growth index, whereas TrkA knockdown impaired the MiaPaCa-2 invasion index and DRG growth ability. Furthermore, TrkA knockdown was also able to partially reverse the promoting effect of NGF overexpression on the MiaPaCa-2 invasion index and DRG growth index (Figure 4(g)–4(h), *p* < 0.01). In a word, these findings reveal that TrkA blockade attenuates NGF induced PC cell migration and neuroinvasion as well as PI3K/Akt/mTOR pathway activation.

### 3.5. NGF Could Upregulate Tumor Derived Exosomal miRNA-21-5p (TDE-miR-21-5p) Expression Level

**Figure 5:**
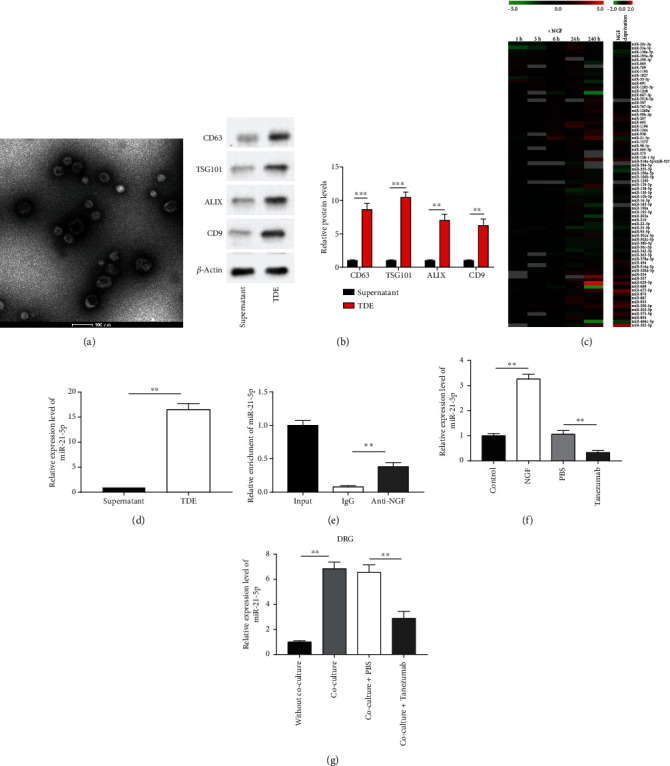
NGF could upregulate TDE-miR-21-5p expression level. (a) Characterization of PC cells-derived exosomes was tested by transmission electron microscopy. Scale bar, 100 nm. (b) The markers of exosomes were tested by western blotting. (c) Heat map analysis of NGF regulated differentially expressed miRNAs in GEO database. (d) RT-qPCR was employed to test miR-21-5p expression in PC cells-derived exosomes. (e) miR-21-5p level in the 3′UTR region of NGF was assessed by RIP assay. (f-g) RT-qPCR was employed to test miR-21-5p expression in MiaPaCa-2 cells treated with NGF or Tanezumab. *N* = 3, ^∗^*P* < 0.05, ^∗∗^*P* < 0.01.

PC cells derived exosomes were collected and cultured for several days. Transmission electron microscopy was used to assess the morphology and size of exosomes extracted from PC cells (Figure 5(a)). Besides, surface markers of exosomes were observed to be significantly expressed in the extracts, including CD9, CD63, TSG101, and Alix (Figure 5(b)). In the GEO dataset we found that miR-21-5p expression was significantly reduced in NGF deficiency (Figure 5(c)). Additionally, we found that miRNA-21-5p expression was highly enriched in PS cells-derived exosomes (Figure 5(d) , *p* < 0.01). A study validated differential miRNA expression by RT-qPCR after NGF treatment and discovered that miR-21-5p level was memorably decreased in NGF deprivation [19]. We further authenticated the binding between NGF and miR-21-5p by using RIP assay (Figure 5(e) , *p* < 0.01). In addition, NGF could promote miR-21-5p, while Tanezumab treatment repressed miR-21-5p levels (Figure 5(f)–5(g), *p* < 0.01).

### 3.6. miR-21-5p Inhibitor Suppressed PNI in PC Cells to Relieve Nociceptive Transmission

**Figure 6:**
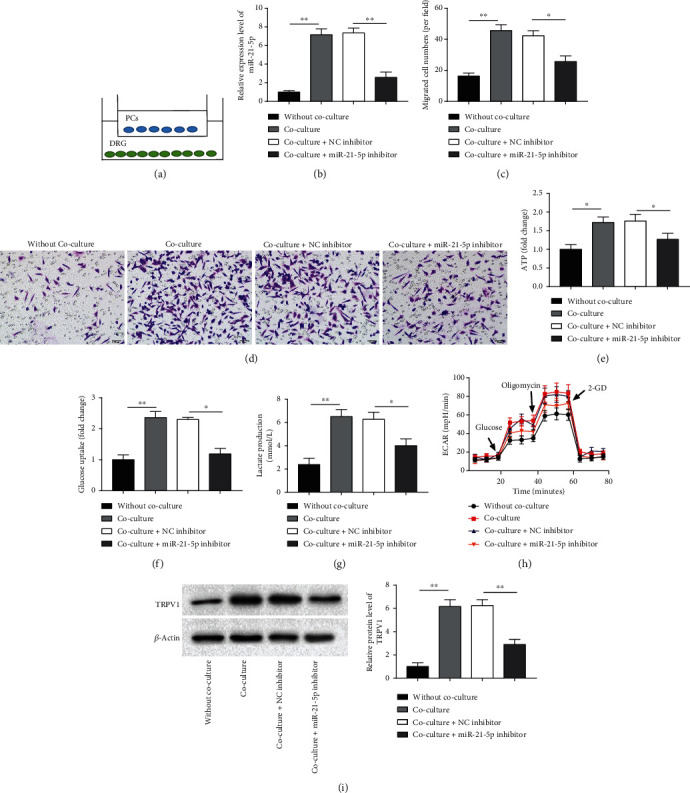
miR-21-5p inhibitor suppressed PNI in PC cells to relieve nociceptive transmission. MiaPaCa-2 cells transfected with miR-21-5p inhibitor were cocultured with DRG cells for 48 h. (a) Diagrammatic sketch of MiaPaCa-2 and DGR cell coculture. (b) RT-qPCR was employed to test miR-21-5p level in DGR cells. (c-d) Transwell was used to detect migration of DGR cells. (e-g) Glucose uptake, lactate production, and ATP assays were employed to test glucose uptake, lactate production, and ATP accumulation. (h) Extracellular acidification rate (ECAR) was employed to test and detect the glycolytic capacity of DGR cells. (i) Western blotting was employed to test TRPV1 expression in DGR cells. *N* = 3, ^∗^*P* < 0.05, ^∗∗^*P* < 0.01.

To further investigate whether miR-21-5p has an effect on PNI in PC cells, MiaPaCa-2 cells were transfected with miR-21-5p inhibitor cocultured with DRG cells.  Figure 6(a) is shown as a coculture system schematic. We discovered that miR-21-5p level, cell migration number, ATP accumulation, glucose absorption, lactate production, and glycolytic capacity, as well as pain state dependent protein TRPV1 expression, were observably increased in DRG cells under coculture conditions, which could be partially reversed by inhibition of miR-21-5p (Figure 6(b)–6(i), *P* < 0.05). Therefore, we hypothesized that TDE-miR-21-5p is an important regulator of PNI in PC cells.

### 3.7. NGF Encouraged PC Cell Neuroinvasive Potential by Activating the Warburg Effect and Upregulating TDE-miR-21-5p Expression

**Figure 7:**
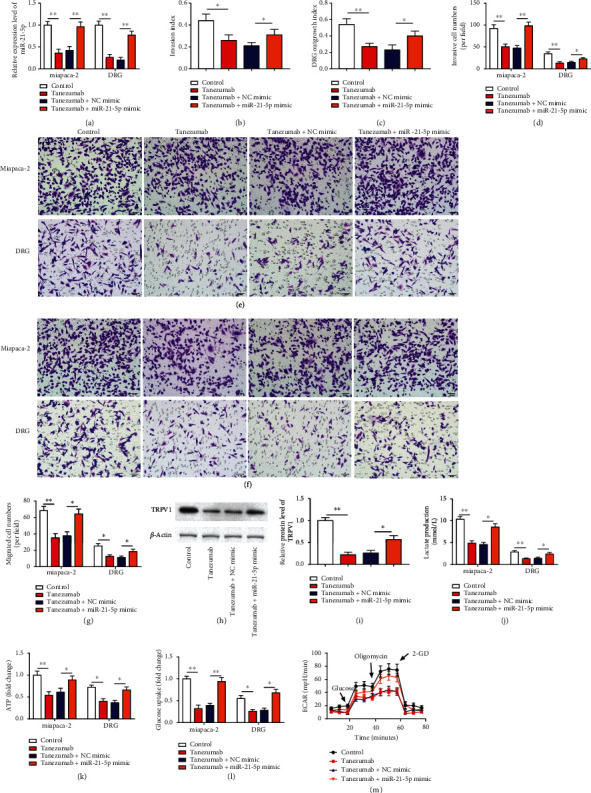
NGF encouraged PC cell neuroinvasive potential by activating the Warburg effect and upregulating TDE-miR-21-5p. Rescue experiments were performed by using cotreatment of 100 ng/ml of Tanezumab and miR-21-5p mimic on MiaPaCa-2 cells for 48 h, followed by coculture with DRG cells. (a) RT-qPCR was employed to test miR-21-5p expression in MiaPaCa-2 and DGR cells cotreatment of Tanezumab and miR-21-5p mimic. (b-c) Neuroinvasion index and DRG growth index are illustrated. (d-g) Transwell was used to detect invasion and migration of MiaPaCa-2 and DGR cells cotreatment of Tanezumab and miR-21-5p mimic. (h-i) Western blotting was employed to test TRPV1 expression in DGR cells. (j-l) Glucose uptake, lactate production, and ATP assays were employed to test glucose uptake, lactate production, and ATP accumulation. (m) Extracellular acidification rate (ECAR) was employed to test and detect the glycolytic capacity of DGR cells. (m) *N* = 3, ^∗^*P* < 0.05, ^∗∗^*P* < 0.01.

To further explore whether NGF and miR-21-5p are functionally relevant in regulating PC, rescue experiments were performed by using cotreatment of Tanezumab and miR-21-5p mimic on MiaPaCa-2 cells, followed by coculture with DRG cells.  Figure 7(a) is presented as the overexpression efficiency of mir-21-5p mimic. Subsequently, intracellular miR-21-5p level also decreased in DRG cells of the Tanezumab-treated MiaPaCa-2 cell group after coculture, and the miR-21-5p level was reversed after transfection of miR-21-5p mimic (Figure 7(a) , *p* < 0.01). Furthermore, Tanezumab treatment significantly decreased the invasion index and DRG growth index of MiaPaCa-2 cells, and the invasion number, cell migration number, ATP accumulation, glucose absorption, lactate production, and glycolytic capacity of MiaPaCa-2 and DRG cells, and TRPV1 protein level in MiaPaCa-2 cells, which were reversed by miR-21-5p upregulation (Figure 7(b)–7(m) , *p* < 0.05). In conclusion, inhibition of miR-21-5p expression in MiaPaCa-2 cells was able to abolish the promoting effect of NGF on PNI in PC cells.

### 3.8. NGF Knockout Markedly Alleviated Nerve Invasion by PC Cells as Well as Relieved Nociceptive Conduction in Animal Models

**Figure 8:**
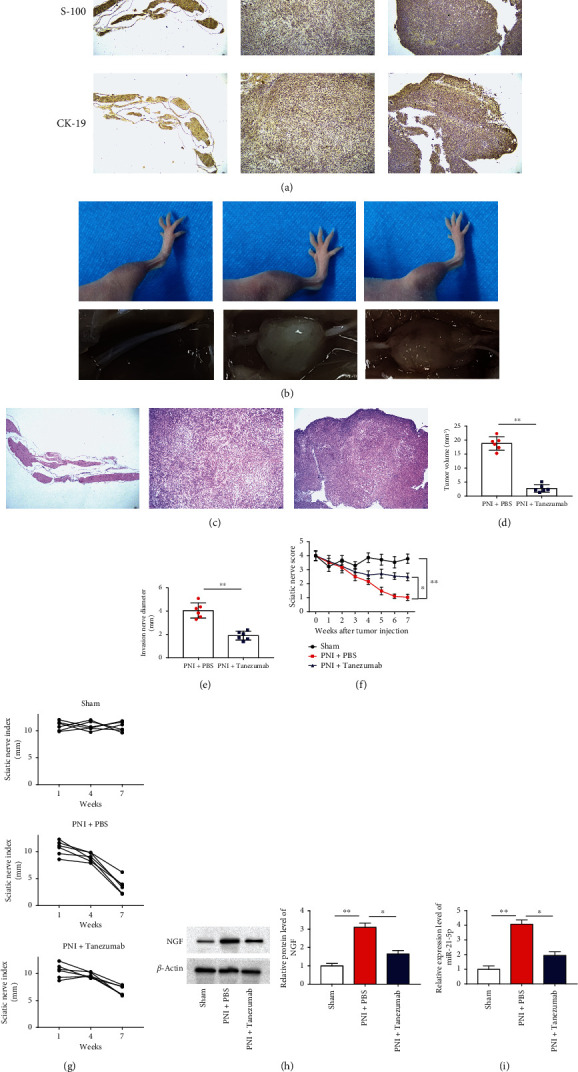
NGF knockout markedly alleviated nerve invasion by PC cells as well as relieved nociceptive conduction in animal models. (a) The expression of S100 (a neural tissue marker) and CK19 (a cancer cell marker) was detected immunohistochemically (scale bar, 1 mm). (b) The mean left sciatic nerve scores and sciatic nerve index in different groups were measured weekly for 7 weeks. (c) Xenograft tumors and corresponding HE staining of orthotopic sciatic nerves from the mouse hindlimbs. (d-g) Tumor volume, invasive nerve diameter, sciatic nerve score, and sciatic nerve index were tested in each group at 7 weeks after injection. (h) Western blotting was employed to test the protein level of NGF in in mouse tumor tissues. (i) RT-qPCR was employed to test the level of miR-21-5p in mouse tumor tissues. *N* = 3, ^∗^*P* < 0.05, ^∗∗^*P* < 0.01.

Subsequently, we used a model of neuroinvasion in nude mice. A total of 15 nude mice were randomly divided into 3 groups: Sham, PNI + PBS, and PNI + Tanezumab. The cell suspension was injected into the middle of the back of the mouse. Eight weeks after injection, tumors were obtained histologically to observe whether the nerves had PNI. The relationship between NGF and PNI was studied by western blot analysis. Using immunohistochemical methods, nerve tissue marker (S100), and tumor marker (CK19) was evaluated in the neural invasion model (Figure 8(a)). Next, mice in the Tanezumab group developed hindlimb dysfunction after 15 days and left hindlimb paralysis by week 7, which was significantly prolonged compared with the PBS group. Seven of the ten mice in the PNI + PBS group became completely paralyzed at week 7, whereas only two mice in the Tanezumab group became paralyzed. All mice in the sham group had normal hindlimb function (Figure 8(b)). Sciatic nerves were excised for HE staining after mice were sacrificed at 8 weeks. HE staining displayed that the diameter of sciatic nerve at the site of primary tumor and 5 mm along the proximal end of sciatic nerve was prominently reduced in PNI + Tanezumab mice compared with PNI + PBS (Figure 8(c)). Tumor volume and invasive nerve diameter were prominently decreased in the Tanezumab group compared with the PNI + PBS control group (Figure 8(d)–8(e)). Sciatic nerve scores and sciatic nerve index were evidently reduced in the PNI + PBS group compared to the sham group, and were back increased after treatment with Tanezumab (Figure 8(f)–8(g)). Further, the results revealed that NGF inhibition could reduce tumor volume and invasive nerve diameter. Additionally, compared with the sham group, PNI + PBS substantially promoted NGF and miR-21-5p expression in mouse tumor tissues, which were dramatically reversed by Tanezumab treatment (Figure 8(h)–8(i)). These data suggest that elimination of NGF can inhibit tumor growth and sciatic nerve invasion into the spinal cord.

## 4. Discussion

PNI is one of the most characteristic biological behaviors of pancreatic cancer and is also one of the leading causes of local recurrence and poor prognosis after curative resection [20]. At present, it is generally accepted that neuroinvasion refers to the existence of reciprocal chemotactic interactions between tumor cells and nerve cells, resulting in the infiltrative growth of nerve bundles with tumor cell extension [21]. In this experiment, Matrigel/DRG and mouse sciatic nerve infiltration models were used to investigate the effect of NGF on the formation of PNI in PC cells and preliminarily explore the paracrine loop of NGF/TrkA/miR-21-5p. Normally, NGF regulates neuronal survival, growth and development, differentiation, and regeneration and functional maintenance [22]. First, we determined NGF expression in PC. NGF level in PC tissues and cell lines was prominently upregulated than in normal tissues and normal pancreatic epithelial cell lines. Besides, NGF promoted MiaPaCa-2 and CFPAC-1 cell invasion and migration, while Tanezumab treatment inhibited cell invasion and migration. Zhu et al. revealed that the mRNA levels of NGF and TrkA in PC were increased by 2.7- and 5.6-fold compared with normal pancreatic tissue, respectively. Moreover, mRNA levels of NGF and TrkA were higher in tumors with perineural invasion than in those without perineural invasion, and perineural invasion was more likely to occur in tumors with high expression of NGF and TrkA, suggesting that NGF released by cancer cells does not act on itself through paracrine or autocrine actions, but rather acts on TrkA presented at the perineurium to bring nerves into contact with cancer cells, which in turn leads to TrkA-mediated perineural invasion [23]. Thus, it illustrates that the NGF/TrkA pathway plays a role in the dissemination and invasion of neural processes along nerves in PC.

In 1920, the German biochemist Warburg discovered that liver cancer cells, in contrast to normal hepatocytes, were more active in their glycolytic activity, leading to the discovery that the programmed metabolism of cancer cells differed from that of other normal cells [24]. Even under oxygen replete conditions, cancer cells metabolize glucose through anaerobic glycolysis to generate the energy needed for cell survival, a metabolic feature of aerobic glycolysis known as the Warburg effect, which is manifested by a high rate of glucose uptake, active glycolysis, and high levels of metabolite lactate [25]. A research found that circ_03955 through miR-3662/HIF-1*α* Axis activation of the Warburg effect functions as a tumor promoter, which may provide a new perspective for PC treatment [26]. ARF6 may contribute to pancreatic cancer development by promoting the Warburg effect [27]. Li et al. [28] displayed that silencing of PKM2 exhibited suppressive effects on pancreatic cancer tumor growth and invasion by altering the Warburg effect. Ye et al. [29] confirmed that lncRNA FEZF1-AS1 made an indispensable contribution to the invasion of pancreatic cancer cells by maintaining the Warburg effect. Additionally, the Warburg effect has been reported to participate in tumor development and also accelerate the neurological diseases. Formaldehyde upregulates the Warburg effect in hippocampal tissue, as evidenced by increased ATP, glucose, and lactate production [30]. Therefore, we speculate that NGF/TrkA signaling may contribute to PC and DRG cell migration and PNI by activating the Warburg effect. TrkA knockdown partially reversed the facilitative effects of NGF on glucose uptake, lactate production, and ATP accumulation. Accumulating evidence has established that the PI3K/AKT/mTOR pathway is one of the key intracellular signaling pathways, and its activation has recently been found to be frequently aberrantly activated in many tumors and plays a critical role in regulating key physiological and pathological cellular processes, including cell proliferation, invasion, cancer progression, and chemoresistance as well as angiogenesis [31]. Currently, many studies have shown that Akt/mTOR signaling promotes the Warburg effect and tumor development by increasing GLUT1 trafficking and activation of glycolytic enzymes, as well as by inducing glycolysis [32]. In this study, we further tested the level of the PI3K/AKT/mTOR pathway proteins under NGF/TrkA pathway stimulation and blockade, and the results showed that PI3K, p-AKT, and p-mTOR protein expression were upregulated in NGF overexpression PC cells, whereas transfection of TrkA siRNA reversed the results.

Exosomes, small extracellular vesicles with a diameter of 30-150 nm that are secreted by almost all living cells in the body and have a phospholipid bilayer resembling the structure of the cell membrane, have been closely followed by researchers in recent years [33]. Exosomes carry a variety of biologically active substances such as DNA, RNA, proteins, liposomes, and metabolic small molecules associated with their cell of origin, which reflect the body's physiopathological state and suggest disease-related biological information, including tumors [34]. Tumor cell or other cells in the tumor microenvironment create a microenvironment suitable for tumor survival and progression by secreting exosomes and transporting tumor related active substances such as DNA, RNA, or proteins into the target cells via exosomes, which in turn regulate the biological functions of the target cells, thereby promoting the occurrence and development of tumors [35]. High baseline miR-21 plasma concentrations are associated with clinical outcomes in cancer patients after induction chemotherapy [36]. Exosomal miR-21-5p could increase the tumor volume, size, and weight of ovarian cancer *in vivo* and promote the development of ovarian cancer [37]. Tang et al. [38] showed that miR-21-5p may encourage cell proliferation, migration, and invasion by disrupting Smad7 expression in lung cancer cells. Besides, high expression of miR-21-5p was demonstrated to be associated with rectal tumor TNM stage and lymph node metastasis [39]. A study using nanomedicines to codeliver miR-21-5p antisense oligonucleotides and gemcitabine for PC treatment showed that the integration of miR-21-5p gene inhibition and gemcitabine treatment by using single-chain variable fragment functionalized nanoparticle carriers exerted synergistic antitumor effects on PC cells [40]. miR-21-5p was obviously increased in PC cells and tissues [41]. Besides, the effect of miR-21-5p on the Warburg effect is positively correlated with the activity of the Wnt/*β*-catenin pathway [42, 43]. The results of this study demonstrate that miR-21-5p is highly enriched in MiaPaCa-2-derived exosomes. We discovered that TDE-miR-21-5p is a considerable regulator of PNI in PC cells. Our results confirmed that NGF was able to upregulate miR-21-5p expression. Furthermore, knocking down the expression of miR-21-5p in MiaPaCa-2 cells was able to abolish the promoting effect of NGF on PNI in PC cells. Because, we speculated that NGF/TrkA promoted neuroinvasion in pancreatic cancer may be associated with upregulation of miR-21-5p by NGF.

As mentioned above, NGF and miR-21-5p levels were prominently overexpressed in PC tissues and cell lines. NGF/TrkA promotes the neuroinvasive potential of PC cells by activating the Warburg effect in DRG cells through upregulation of TDE-miR-21-5p expression. Collectively, this study demonstrates that a paracrine loop between PC cells and the DRG mediated by NGF/TrkA/miR-21-5p contributes to PNI.

## Data Availability

The datasets used during the present study are available from the corresponding author on reasonable request.
